# Selective right heart valve remodelling in a mouse model of carcinoid disease revealed by high-resolution episcopic microscopy

**DOI:** 10.1038/s41598-025-16064-8

**Published:** 2025-08-30

**Authors:** Gaspard Suc, Mustafa Habib, Sofiane Mohammed, Gregory Franck, Samuel Sitbon, Jacques Callebert, Marine Jullien, Baptiste Bazire, Philippe Ruszniewski, Louis de Mestier, Audrey Cailliau, Lydia Deschamps, Dimitri Arangalage, Antonino Nicoletti, Giuseppina Caligiuri, Jamila Laschet

**Affiliations:** 1 Paris Cité University and Sorbonne Paris Nord University, INSERM, LVTS, 75018 Paris, France; 2 Paris Cité University and Sorbonne Paris Nord University, INSERM, LVTS, Cardiology Department, AP-HP, Bichat Hospital, 75018 Paris, France; 3 Paris Cité University, INSERM U942, Biochemistry and Molecular Biology Department, AP-HP, Lariboisière Hospital, 75010 Paris, France; 4 Paris Cité University, Pancreatology and Digestive Oncology Departement, AP-HP, Beaujon Hospital, 92110 Clichy, France; 5 Paris Cité University and Sorbonne Paris Nord University, INSERM, LVTS, Pathological Anatomy and Cytology Department, AP-HP, Bichat Hospital, 75018 Paris, France

**Keywords:** Serotonin, Heart valves/pathology, High-resolution episcopic microscopy, Animal experimentation, Mice, Cardiovascular diseases, 3-D reconstruction, Valvular disease, Animal disease models, Super-resolution microscopy

## Abstract

Carcinoid heart disease, a severe complication of neuroendocrine tumors, affects up to 50% of patients and is challenging to treat due to a limited understanding of its mechanisms. The disease is characterized by structural remodeling and thickening of the right heart valves, associated with elevated levels of serotonin (5-HT) released from tumor cells that have spread to the liver. Existing animal models have limitations as they either use mice with compromised immune systems or employ methods that don’t consistently evaluate valve changes. We developed an improved experimental model by implanting syngeneic liver-targeted melanoma cells that were genetically engineered to produce 5-HT through the expression of the enzyme tryptophan hydroxylase type 1 (Tph1) in wild-type C57BL/6 mice. We introduced high-resolution episcopic microscopy (HREM) for comprehensive assessment of valve pathology and morphometry. Five weeks after implantation, mice exhibited increased 5-HT/creatinine urinary level ratios and HREM imaging showed selective thickening and structural remodeling of right heart valves (tricuspid and pulmonary), correlating with 5-HT/creatinine urinary level ratio, while left heart valves remained unaffected. Our data suggest that this non-immunosuppressed, right-heart valve restricted model reproduces key features of human carcinoid heart disease and, combined with HREM analysis, provides a valuable platform for studying disease mechanisms and testing potential therapies.

**Supplementary Information:**

The online version contains supplementary material available at 10.1038/s41598-025-16064-8.

## Introduction

Carcinoid heart disease affects up to 50% of patients with neuroendocrine tumors and is characterized by severe changes in the right heart valves^1,2^. These changes are primarily driven by 5-HT, a signaling molecule released from tumor cells that have spread to the liver. Despite its significant impact on patient survival, no drug therapy has proven effective in stopping the progression of carcinoid heart disease, largely due to incomplete understanding of how the disease begins and develops^1–4^.

Examining the heart valves affected by carcinoid disease presents unique challenges. Most studies have looked at valves already severely damaged, typically obtained during valve replacement surgeries^4,5^. This limits our understanding of how the disease starts and progresses in its early stages. This knowledge gap has prompted researchers to develop experimental models using laboratory animals^6^.

However, existing animal models have significant limitations^7–11^. Some approaches, such as injecting 5-HT under the skin in rats^8^ or giving it orally to rabbits^10^, cause widespread damage throughout the heart rather than the specific valve changes seen in human patients. While implanting human neuroendocrine tumor cells in the liver of mice with suppressed immune systems better mimics the disease mechanism^9^, the compromised immune system limits how well these findings can be applied to human patients who have normal immune function. To overcome these limitations, we developed a model using genetically modified syngeneic mouse melanoma cells developing in the liver, aiming to replicate the pathophysiological sequence of carcinoid syndrome where hepatic metastases expose right heart valves to elevated 5-HT levels. The hepatic venous system provides a direct vascular link between hepatic tumors and the right heart. Unlike systemic circulation, blood from the liver drains directly into the inferior vena cava via the hepatic veins, bypassing the liver metabolic filtration that, together with pulmonary metabolic pathways, normally degrade circulating amine-derived hormones^12^. This anatomical configuration allows vaso- and/or neuro-active hormones, such as 5-HT or other neuroendocrine tumor-derived factors from liver metastases, to reach the right atrium and right ventricle in higher concentrations compared to the left heart (due to pulmonary metabolism), potentially explaining the right heart restriction of carcinoid fibrotic remodeling and valvular dysfunction. By leveraging this hepatic-cardiac connection, our model enables the study of localized hepatic tumor secretion and its direct impact on right heart function, closely mimicking the clinical progression of carcinoid heart disease. This approach provides a physiologically relevant setting to investigate cardiac-specific effects of hepatic tumor activity, overcoming the limitations of massive systemic 5-HT exposure and immune constraints in prior models.

A major technical challenge in studying carcinoid heart disease is accurately measuring valve changes, a task that becomes even more difficult in small‑animal models such as mice. Heart valves have complex three-dimensional structures, particularly the tricuspid valve on the right side of the heart, making standardized evaluation difficult using conventional methods. Ultrasound imaging in mice is challenging for right heart structures; even ultrafast echocardiography with cutting-edge machines does not allow visualization of the right valves in 2D, and only Doppler measurements can be performed^13^, which could only be useful to assess moderate to severe regurgitation in the latest stages of the disease but not in its initiation. Furthermore, traditional tissue sectioning cannot precisely control valve orientation during processing, leading to inconsistent results. High-resolution episcopic microscopy (HREM) offers a promising solution for complex valve assessment. While this technique has been established in studying embryo development and cancer^14^, it has not yet been applied to adult mouse heart valve research. Yet, HREM allows generation of detailed, three-dimensional reconstructions of cardiac structures while maintaining their precise spatial relationships, potentially overcoming the limitations of conventional imaging methods.

Our objective was to develop a mouse model with a normal immune system using liver-targeted, 5-HT-producing melanoma cells to replicate the selective right heart valve involvement seen in patients. Additionally, we sought to validate HREM as a tool for standardized, comprehensive assessment of valve changes in this model.

## Results

### Validation of 5-HT production

**Fig. 1:**
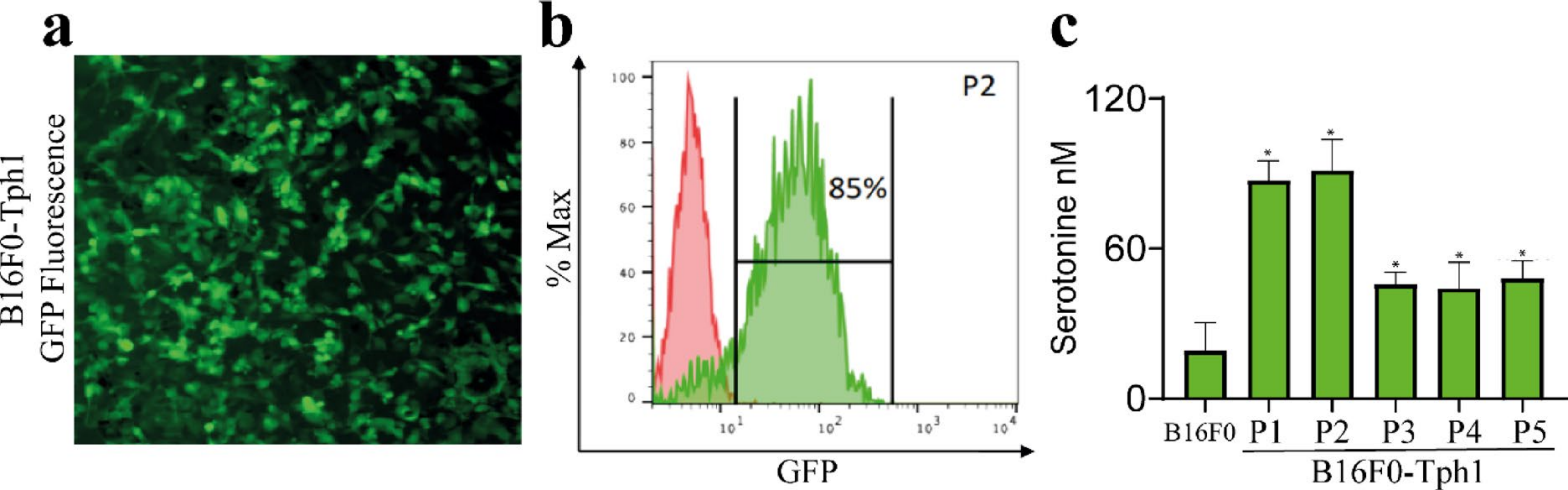
Validation of B16F0-Tph1 cell line. (**a**) Representative fluorescence microscopy image showing GFP expression in B16F0-Tph1 cells. (**b**) Flow cytometry analysis demonstrating 85% GFP-positive cells at passage 2 (P2). Red histogram: non-modified B16F0 cells; green histogram: B16F0-Tph1 cells. (**c**) 5-HT concentration in culture supernatants showing sustained production in B16F0-Tph1 cells across passages (P1-P5) compared to B16F0 controls. Values represent median ± IQR from n = 3 independent experiments per condition. Statistical significance was determined using Kolmogorov–Smirnov test (*p < 0.05).

In vitro characterization demonstrated successful genetic modification of B16F0 cells. Fluorescence microscopy and flow cytometry confirmed high efficiency of Tph1-GFP transduction, with 85% of cells expressing GFP at passage 2 (Fig. 1a,b). Functional validation showed robust 5-HT production in B16F0-Tph1 cells compared to non-modified controls. B16F0-Tph1 cells produced significantly more serotonin across all passages compared to B16F0 controls: P1 (88.00 ± 14.75 vs 19.00 ± 20.90 nM, *p* = 0.0286), P2 (90.50 ± 23.30 vs 19.00 ± 20.90 nM, *p* = 0.0286), P3 (44.00 ± 8.25 vs 19.00 ± 20.90 nM, *p* = 0.0286), P4 (43.50 ± 18.50 vs 19.00 ± 20.90 nM, *p* = 0.0286), and P5 (46.00 ± 12.50 vs 19.00 ± 20.90 nM, *p* = 0.0286). While 5-HT production decreased in later passages, it remained significantly elevated through to passage 5 (Fig. 1c).

### Model development and optimization

In the first pilot experiment evaluating tumor burden and survival with varying cell numbers (500–1000–2500–10,000 cells, n = 2 per group), all mice receiving ≥ 2500 cells (n = 4) showed extensive peritoneal carcinomatosis and were euthanized.

**Fig. 2:**
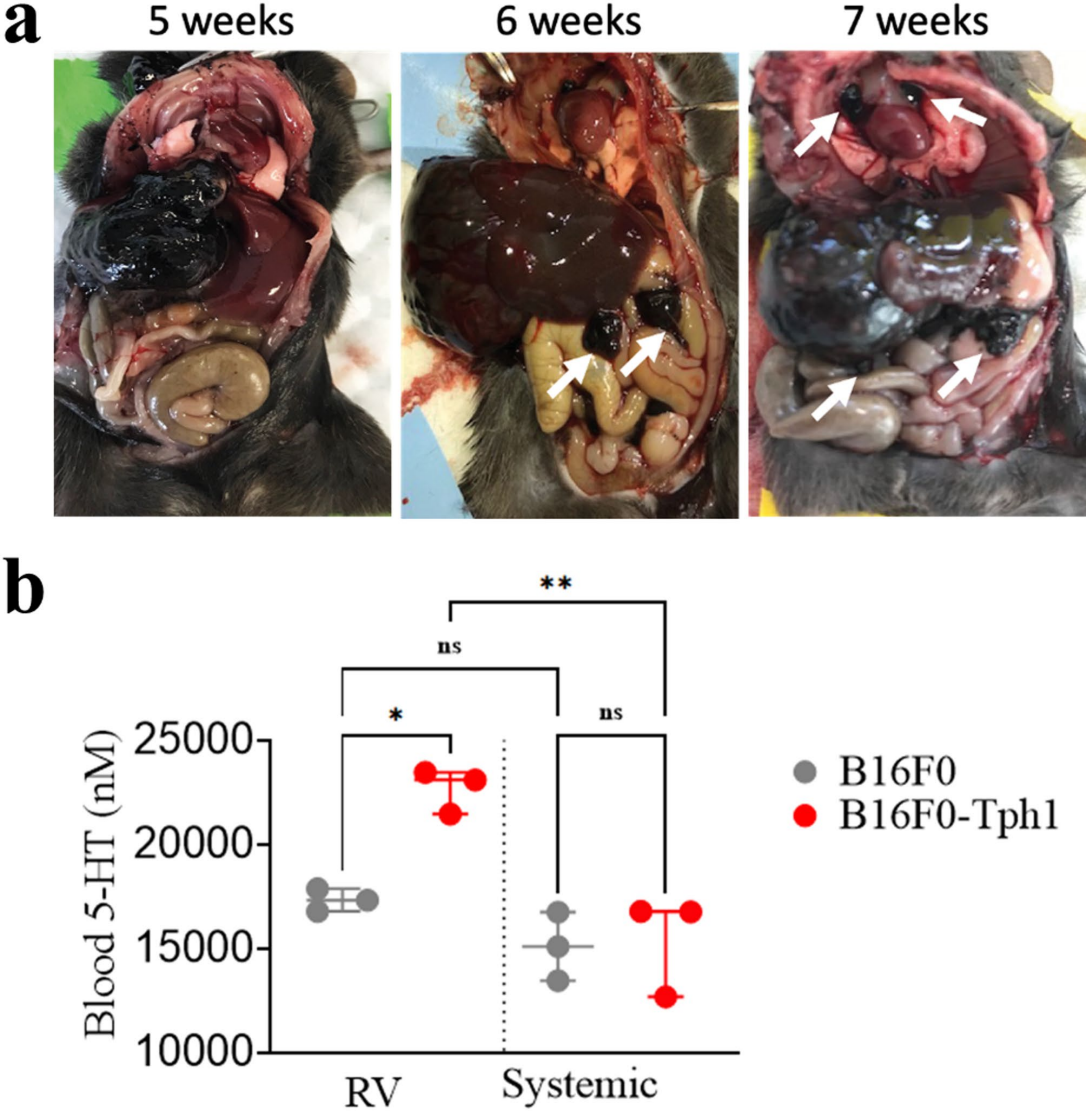
Macroscopic dissemination of tumor cells over time and blood 5-HT elevation in RV following intrahepatic B16F0-Tph1 implantation. (**a**) Representative images of mice injected with 1000 B16F0-Tph1 cells in the liver, showing progression of tumor spread at 5, 6 and 7 weeks post-injection. White arrows indicate extrahepatic dissemination (peritoneal carcinomatosis at 6 weeks and mediastinal lymph node metastases at 7 weeks). No extrahepatic dissemination was detected at 5 weeks, establishing this timepoint as optimal for the main study. (**b**) Blood 5-HT concentrations measured in the right ventricle (RV) and systemic circulation 5 weeks after intrahepatic implantation in B16F0-Tph1 and B16F0 mice. Data are presented as individual points with median ± IQR; n = 3 per group. Statistical significance was determined using Kolmogorov–Smirnov test (*p < 0.05 and **p < 0.01).

The second pilot determined optimal exposure duration using 1000 cells (n = 3 per group). The melanin pigmentation of B16F0 cells enabled clear macroscopic identification of tumor spread. At 5 weeks, mice showed tumors exclusively in the liver, with no secondary spread to the lungs or other organs. Beyond 6 weeks, peritoneal carcinomatosis and mediastinal lymph node metastases developed (Fig. 2a).

At 5 weeks, blood 5-HT concentration was significantly increased in the right ventricle (RV) of B16F0-Tph1 mice compared to B16F0 mice (23,098 ± 2000 vs 17,324 ± 1082 nM, *p* = 0.0127), and significantly higher in RV compared to systemic circulation in B16F0-Tph1 mice (23,098 ± 2000 vs 16,755 ± 4104 nM, *p* = 0.0020). No significant differences were observed between RV and systemic circulation in B16F0 mice (17,324 ± 1082 vs 15,099 ± 3286 nM, *p* = 0.3612), or between systemic blood of the two groups (16,755 ± 4104 vs 15,099 ± 3286 nM, *p* = 0.9947) (Fig. 2b). These results indicate a selective exposure of the right heart to elevated 5-HT levels in the B16F0-Tph1 model, consistent with the clinical presentation of carcinoid syndrome.

**Fig. 3:**
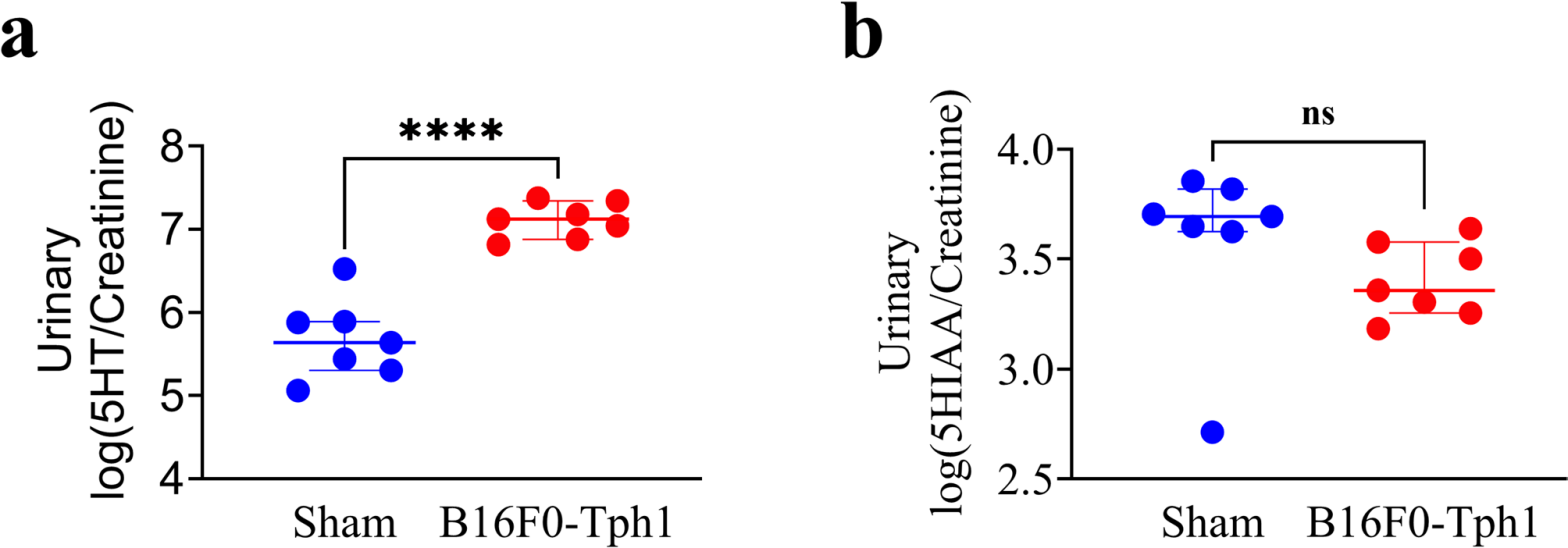
Analysis of urinary 5-HT and metabolite. (**a**) Log-transformed urinary serotonin (5-HT/creatinine) levels in sham (n = 7) and B16F0-Tph1 (n = 7) mice at 5 weeks post-surgery. (**b**) Log-transformed urinary 5-hydroxyindoleacetic acid (5-HIAA/creatinine) levels in the same groups. Data are shown as individual points with median ± IQR. Statistical significance was determined using Kolmogorov–Smirnov test (****p < 0.0001, ns: not significant).

In the main experiment, the B16F0-Tph1 group (n = 10) experienced two spontaneous deaths: one occurring 24 h post-surgery due to internal bleeding, and another on day 29 from tumor progression. In the sham group (n = 10), all mice survived, though urine collection was not possible from two mice. One heart per group was excluded from HREM analysis due to technical artifacts (air bubbles in the specimen block). Mice injected with non-modified B16F0 cells exhibited significantly higher mortality (80%) compared to those receiving B16F0-Tph1 cells (20%). In this experiment, we did not collect blood from the right intraventricular cavity to preserve the heart for HREM analyses. Thus, to comprehensively characterize our model’s 5-HT production and metabolism, we analyzed both urinary 5-HT and its metabolite 5-HIAA. Only two mice from B16F0 group survived by the time of sacrifice. Their hearts were kept and used for HREM analysis, but unfortunately, we were unable to collect their urine. Five weeks after cell implantation, B16F0-Tph1 mice exhibited significantly higher log-transformed urinary 5-HT/creatinine ratios compared to sham controls (7.12 ± 0.46 vs 5.64 ± 0.59, *p* < 0.0001) (Fig. 3a). In contrast, urinary 5-HIAA/creatinine ratios were similar between B16F0-Tph1 mice and sham controls (3.69 ± 0.19 vs 3.36 ± 0.32, *p* = 0.2962) (Fig. 3b).

### Valve morphology and remodelling assessment

HREM analysis revealed morphological patterns consistent with carcinoid heart disease in B16F0-Tph1 mice. Right heart valves in B16F0-Tph1 mice exhibited characteristic nodular thickening with spatial heterogeneity in both tricuspid and pulmonary valves features typical of carcinoid-induced valve remodelling compared to sham valves.

**Fig. 4:**
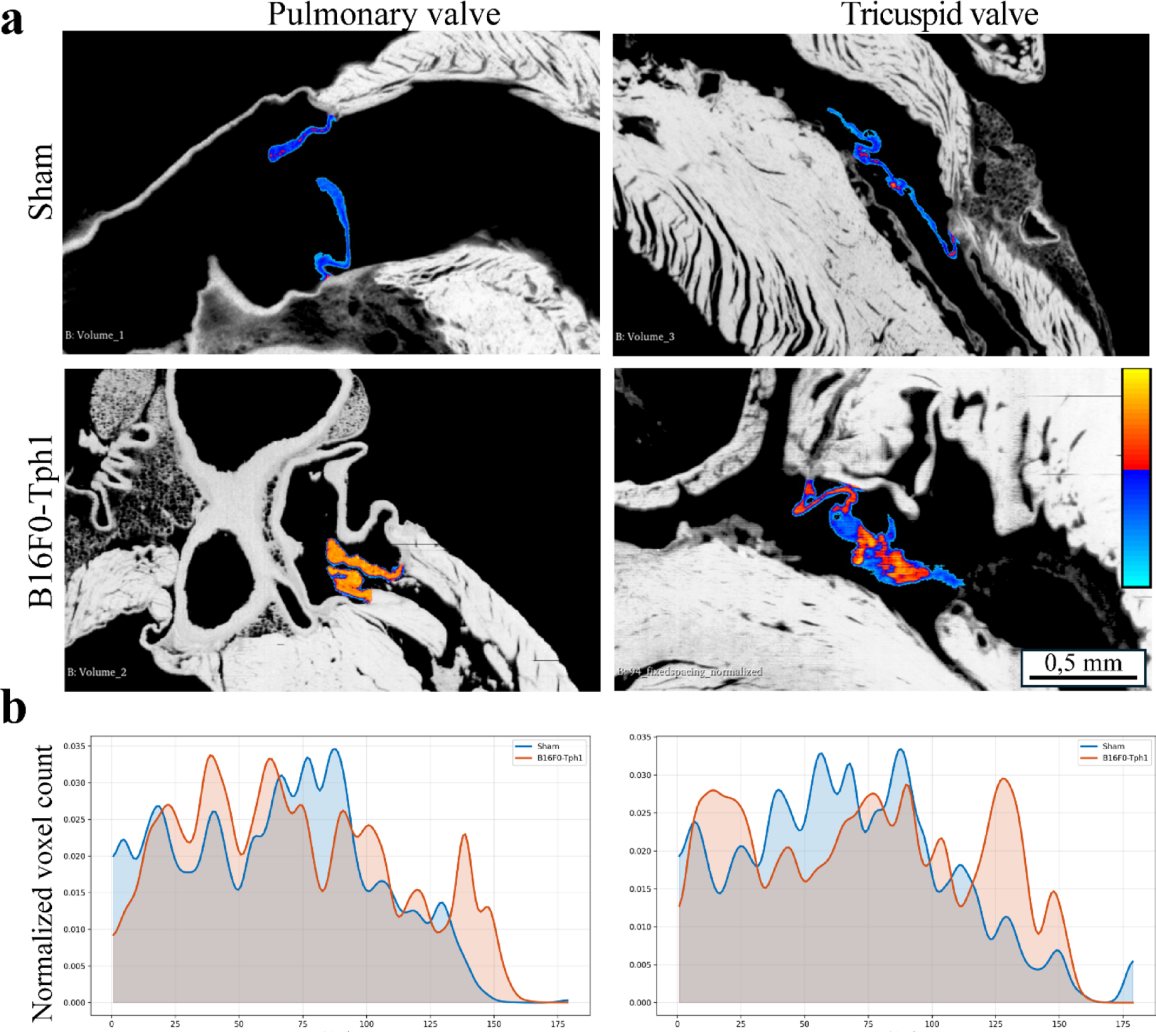
Heterogeneous signal intensity distribution in pulmonary and tricuspid valves of B16F0-Tph1 mice. (**a**) Representative HREM images with grayscale intensity overlays (blue: low intensity; yellow: high intensity). Sham valves show uniform low-intensity signal, while B16F0-Tph1 valves exhibit spatially heterogeneous intensity patterns, with distinct high-intensity regions adjacent to low-intensity zones. (**b**) Normalized intensity histograms demonstrate a rightward shift in B16F0-Tph1 mice, with new high-intensity voxel populations (120–180 range) absent in sham controls. Scale bar: 0.5 mm.

Analysis of HREM-derived grayscale intensity revealed pronounced spatial heterogeneity of grayscale signal intensity within the tricuspid valves of B16F0-Tph1 mice (Fig. 4a). Unlike the uniform low-intensity signal in Sham controls, B16F0-Tph1 valves contained intermixed regions of low (blue) and high (orange/yellow) intensity, reflecting localized differences in the optical properties of the valve tissue.

Quantification of intensity distributions (Fig. 4b). confirmed a significant increase in high-intensity voxels (120–180 range) in B16F0-Tph1 mice, absent in Sham. This shift in intensity distribution indicates a higher degree of spatial heterogeneity in the optical properties of the valve tissue in B16F0-Tph1 mice. Importantly, HREM signal intensity does not directly measure tissue density or extracellular matrix composition, and further histological validation is required to determine the nature of these structural changes.

**Fig. 5:**
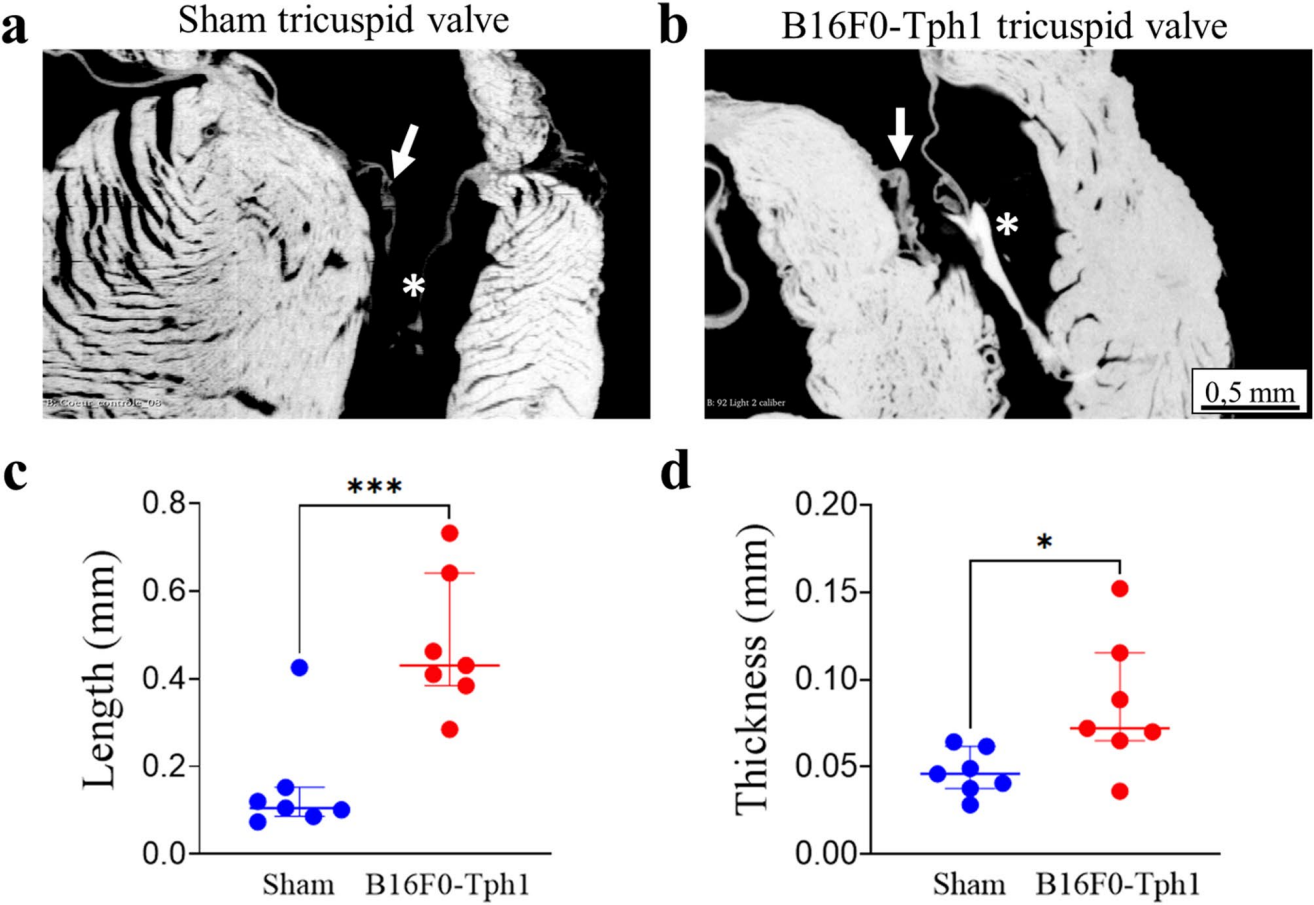
High-resolution episcopic microscopy (HREM) analysis reveals carcinoid-induced pathology in the tricuspid valve subvalvular apparatus of B16F0-Tph1 mice. Representative HREM images of the tricuspid valve (white arrow) and associated chordae tendineae (white asterisk) in Sham (**a**) and B16F0-Tph1 (**b**) mice. Quantitative analysis shows increased chordae tendineae length (**c**) and thickness (**d**) in B16F0-Tph1 compared to Sham mice. Data are shown as individual points with median ± IQR; n = 7 per group. Statistical significance was determined using Kolmogorov–Smirnov test (***p < 0.0001, *p < 0.05, ns: not significant). Scale bar: 0.5 mm.

HREM analysis reveals also carcinoid-induced pathology in the tricuspid valve and subvalvular apparatus of B16F0-Tph1 mice compared to Sham (Fig. 5). The tricuspid valve of B16F0-Tph1 mice displayed significant leaflet thickening compared to Sham valve (Fig. 5a,b). This was accompanied by marked changes in chordae tendineae which were significantly elongated (0.43 ± 0.26 vs 0.11 ± 0.07, *p* = 0.0009) (Fig. 5c) and thickened (0.07 ± 0.05 vs 0.05 ± 0.02, *p* = 0.0248) (Fig. 5d).

**Fig. 6:**
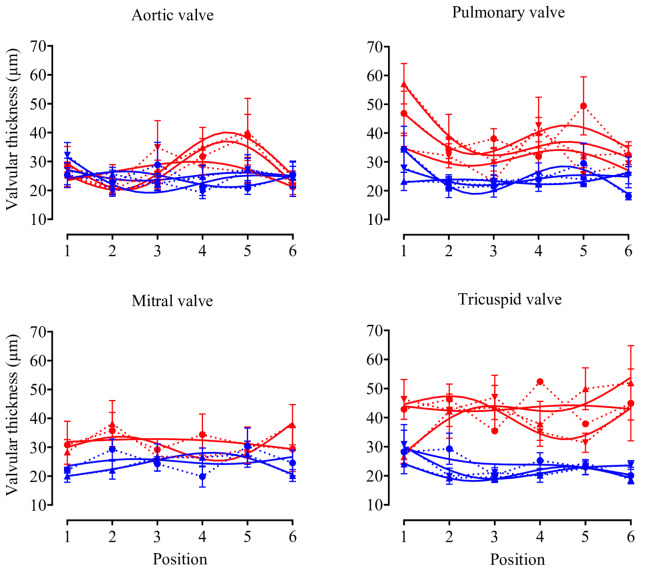
Cardiac valve thickness patterns. Valve thickness was measured at 6 equidistant points from free edge (position 1) to base (position 6) in B16F0-Tph1-treated mice (red lines) and sham controls (blue lines) in all cusps and leaflets (triangle = anterior, circle = posterior or left, inverted triangle = septal or right). Solid lines are spline curves of actual data (dotted lines). Data are presented as median ± IQR; n = 7 per group.

Valve thickness measurements at equidistant points from free edge to base revealed consistent patterns across the valve surface (Fig. 6). The absence of significant position-dependent effects allowed pooling of measurements for statistical analysis.

**Fig. 7:**
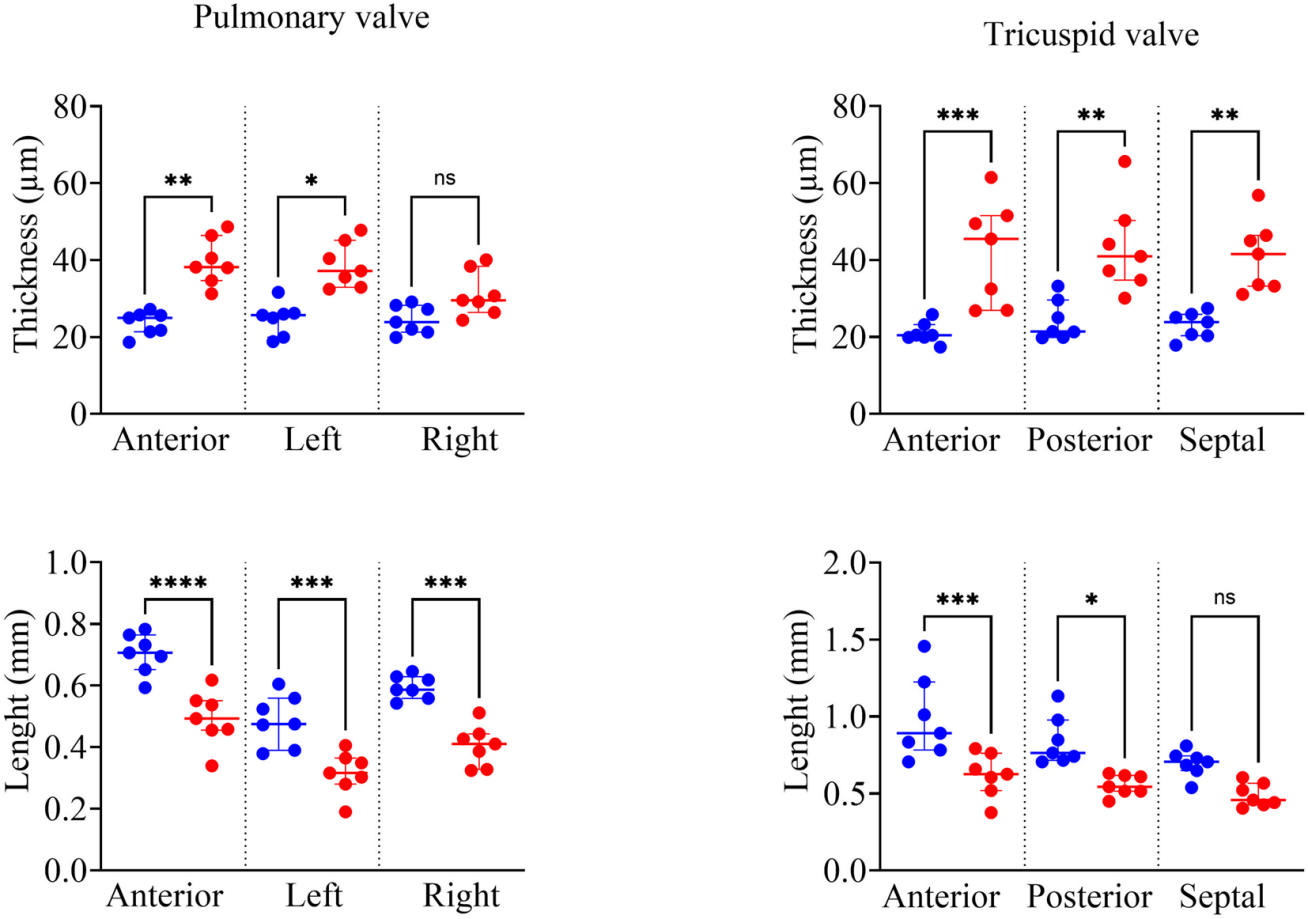
Valvular thickness and length analysis. Comparison between B16F0-Tph1 mice (red dots) and sham controls (blue dots) across different valve leaflets and cusps. Given the absence of position effect (Fig. 6), the six measurements along each leaflet/cusp were used as repeated measures. Tricuspid valve showed significant thickening across all cusps, while pulmonary valve demonstrated significant thickening in anterior and left cusps but not the right cusp. Both tricuspid and pulmonary valves showed decreased length in anterior cusps, with additional reductions in posterior (tricuspid) and left (pulmonary) cusps. Data are presented as median ± IQR; n = 7 per group. Statistical significance was determined using Kolmogorov–Smirnov test (***p < 0.0001, **p < 0.0001, *p < 0.0001, ns: not significant).

Valve morphometric analysis revealed distinct remodeling patterns of right-sided valves (Fig. 7) characterized by both thickening and shortening. Tricuspid valve thickness increased uniformly across all cusps: anterior (45.50 ± 24.60 vs 20.41 ± 3.35, *p* = 0.0009), posterior (40.95 ± 15.51 vs 21.44 ± 9.71, *p* = 0.0030), and septal (41.53 ± 13.21 vs 23.89 ± 5.57, *p* = 0.0051). Pulmonary valve changes were heterogeneous, with significant thickening in anterior (38.15 ± 11.76 vs 24.97 ± 4.35, *p* = 0.0035) and left cusps (37.22 ± 12.23 vs 25.66 ± 6.18, *p* = 0.0220), while the right cusp remained unaffected (29.53 ± 12.01 vs 23.86 ± 6.98, *p* > 0.9999). Left-sided valves showed no structural alterations, with aortic and mitral valve thickness remaining comparable between groups (Supplementary Figure 1).

Concurrent with thickening, valve leaflet shortening was observed in both right-sided valves. Tricuspid valve length in B16F0-Tph1 decreased selectively in anterior (0.63 ± 0.24 vs 0.89 ± 0.44, *p* = 0.0007) and posterior cusps (0.54 ± 0.10 vs 0.76 ± 0.26, *p* = 0.0122), with the septal cusp preserved (0.46 ± 0.14 vs 0.71 ± 0.10 *p* = 0.1326). Pulmonary valve demonstrated more extensive shortening, affecting anterior (0.49 ± 0.10 vs 0.71 ± 0.11, *p* < 0.0001), left (0.32 ± 0.09 vs 0.48 ± 0.17, *p* = 0.0008), and right cusps (0.41 ± 0.12 vs 0.59 ± 0.07 *p* = 0.0002)."

### Volumetric analysis of cardiac valves

**Fig. 8:**
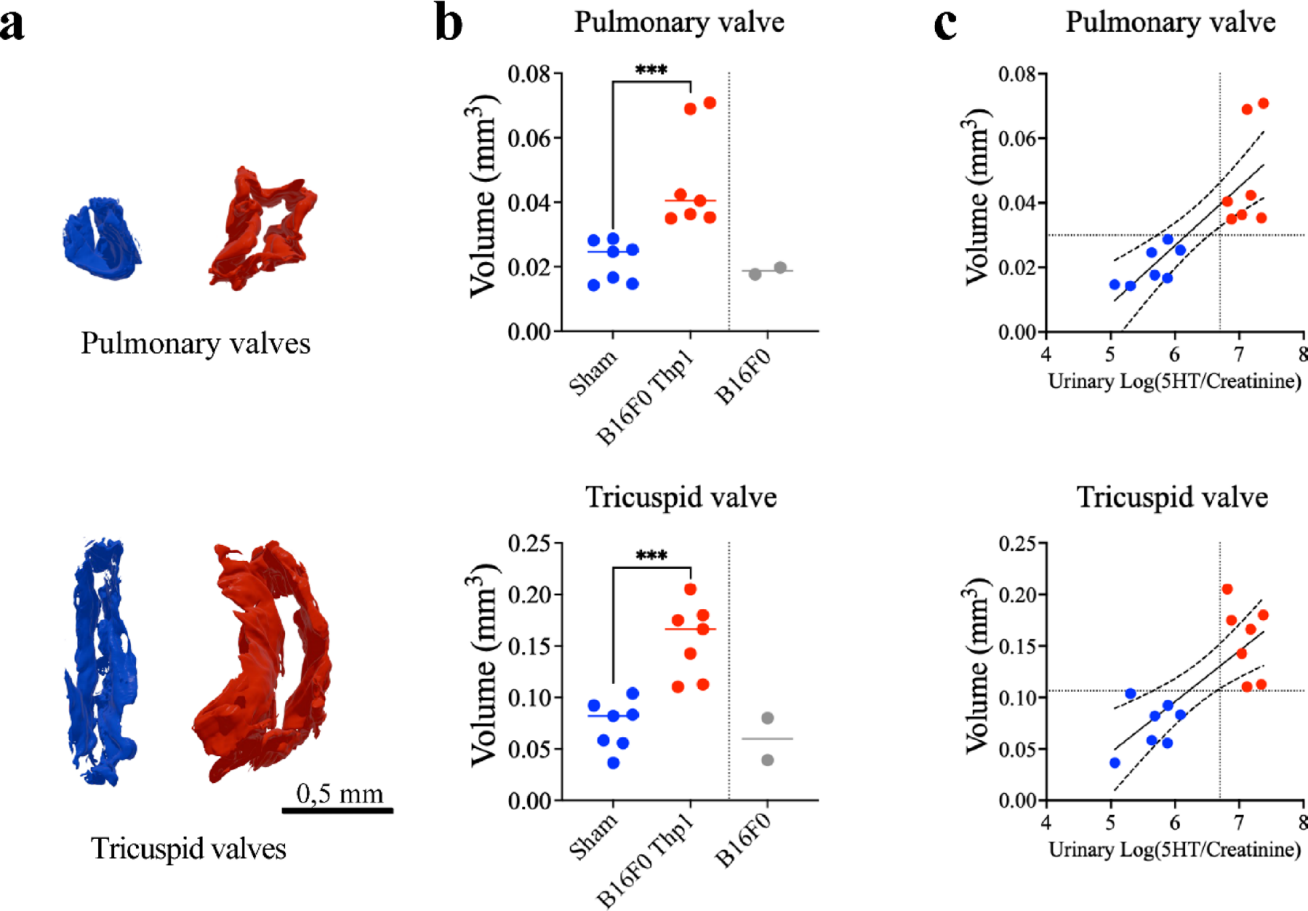
Volumetric analysis and correlation of cardiac valves with urinary 5-HT. (**a**) 3D rendering examples of measured volumes for pulmonary and tricuspid valves in sham (blue) and B16F0-Tph1 (red) mice. (**b**) Quantitative comparison of valve volumes showing significantly increased volumes in B16F0-Tph1 mice (n = 7) compared to sham (n = 7) and B16F0 controls (n = 2) for both pulmonary and tricuspid valves. Data are presented as median ± IQR. (**c**) Linear regression analysis showing the relationship between urinary log(5-HT/creatinine) and valve volume (mm^3^) in tricuspid valve (left) and pulmonary valve (right). Blue dots represent sham animals and red dots represent B16F0-Tph1 animals. Solid lines indicate linear regression with 95% confidence intervals (dashed lines). Dotted vertical and horizontal lines represent threshold values calculated using stepwise regression, yielding high diagnostic performance (pulmonary valve: 86% sensitivity and specificity; tricuspid valve: 100% sensitivity and specificity) for detecting pathological valve thickening. Values are presented as median ± IQR. Scale bar: 0.5 mm. Statistical significance was determined using one-way ANOVA followed by Tukey’s multiple comparisons test (***p < 0.001; ns, not significant).

Three-dimensional rendering of heart valve architecture through comprehensive HREM imaging enabled precise volumetric quantification of valve remodeling (Fig. 8a,b). Mice bearing B16F0-Tph1 tumors exhibited substantial valvular enlargement compared to sham controls, with pulmonary valve volumes expanding 2-fold (0.04 ± 0.03 versus 0.02 ± 0.01, *p* = 0.0006) and tricuspid valve volumes increasing 2.1-fold (0.17 ± 0.07 versus 0.08 ± 0.04, *p* = 0.0006). Importantly, examination of the two surviving mice bearing non-modified B16F0 tumors revealed no appreciable valve alterations despite tumor presence, though limited sample size precluded formal statistical analysis. These findings demonstrate that pathological valve remodeling is specifically dependent on serotonin overproduction rather than tumor burden alone. In contrast to right heart valves, mitral and aortic valve volumes were unchanged between B16F0-Tph1 and sham groups (Supplementary Figure 9).

### Correlation between 5-HT exposure and valve remodelling

To examine the relationship between serotonin production and valve remodeling, we analyzed correlations between the detected urinary 5-HT trace levels (log-transformed urinary 5-HT/creatinine ratios) and right heart valve volumes. Figure 8 c shows these correlations across all experimental animals. Linear regression analysis revealed significant positive correlations for both tricuspid (R^2^ = 0.59, *p* = 0.0014) and pulmonary (R^2^ = 0.72, *p* = 0.0001) valves, with evidence of threshold effects observed at approximately log(5-HT/creatinine) values of 6.5–7.0, above which valve volumes increased more dramatically.

## Discussion

Several experimental models have attempted to replicate carcinoid heart disease, but they face significant limitations. Systemic 5-HT administration leads to non-selective cardiac damage affecting both left and right heart structures^7,10^, failing to reproduce the right-sided predominance characteristic of the human disease. Models using human neuroendocrine tumor cells require immunodeficient mice^11^, limiting their translational relevance. Our study addresses these constraints through methodological innovations that provide new insights into disease pathophysiology. We developed an immunocompetent mouse model that selectively affects right heart valves, accurately mirroring the human disease pattern. This approach using genetically modified melanoma cells offers several unique advantages over previous models. The liver-targeted delivery of 5-HT-producing cells replicates the anatomical and physiological features of carcinoid syndrome, where hepatic metastases expose right heart valves to elevated 5-HT levels while sparing left heart structures after pulmonary metabolism^2–4^.

The intricate three-dimensional architecture of cardiac valves poses additional methodological challenges for quantitative assessment, particularly in murine models^13,15,16^. Conventional approaches such as two-dimensional echocardiography^13,16^ and standard histological sectioning inherently struggle with consistent valve orientation and sampling, potentially missing regional variations in valve pathology due to plane-of-section artifacts^17,18^. Our implementation of high-resolution episcopic microscopy (HREM) addresses these limitations by enabling standardized, spatially-resolved analysis of entire valve structures with micrometer precision^14,17^. This technique revealed unprecedented details of valve remodeling beyond conventional thickness measurements, demonstrating characteristic pathological features including increased grayscale signal intensity, valve leaflet retraction, and extensive remodeling of the subvalvular apparatus. The comprehensive three-dimensional reconstruction of all cardiac valves while preserving their spatial relationships allowed standardized measurements regardless of heart orientation during embedding^14,18^, overcoming a crucial methodological barrier that has limited precise morphometric analysis in previous animal models^19,20^. These structural changes likely contribute to valve dysfunction and are consistent with the morphological features observed in human disease, while revealing subtle patterns of differential remodeling that might otherwise remain undetected. However, we do not claim direct evidence of fibrosis or calcification based on HREM data alone.

The differential patterns of valve thickening observed in our model provide insights into potential biomechanical factors that may contribute to serotonin-mediated valve pathology. While the tricuspid valve exhibited homogeneous thickening across all leaflets, the pulmonary valve demonstrated heterogeneous involvement with significant changes in anterior and left cusps but relative sparing of the right cusp. This spatial heterogeneity aligns with findings from Balachandran et al.^21,22^, who demonstrated that cyclic stretch not only upregulates 5-HT2A and 5-HT2B receptor expression in valve tissue but also enhances serotonin responsiveness through mechanosensitive pathways.

Particularly relevant to our observations is Balachandran’s discovery that the 5-HT(2A) receptor subtype is uniquely sensitive to elevated stretch, with 5-HT(2A) antagonists reducing collagen synthesis, cell proliferation, and tissue stiffness under both normal and elevated stretch conditions^22^. The heterogeneous involvement of pulmonary valve cusps we observed could potentially reflect different mechanical stress distributions across the semilunar valves, with regions experiencing greater cyclic stretch exhibiting enhanced 5-HT(2A) mechanosensitivity and consequently more pronounced thickening. In contrast, the more uniform thickening pattern in the tricuspid valve might suggest a different mechanical environment or potentially different receptor subtype distributions between atrioventricular and semilunar valves^21^.

This interaction between biomechanical stimuli and serotonin signaling suggests that valve pathology in carcinoid syndrome results from a complex interplay between circulating serotonin levels, local valve exposure, and mechanosensitive amplification of serotonin receptor signaling^22^. This interpretation aligns with clinical observations in carcinoid patients, where elevated serum serotonin shows high sensitivity (100%) but poor specificity (46%) for heart valve disease with no clear diagnostic cutoff^23^, whereas NT-proBNP, a marker of cardiac strain, often correlates better with carcinoid heart disease severity than systemic serotonin measurements^24,25^. These clinical findings suggest that local tissue effects of serotonin may be more important than systemic concentrations in determining valvular pathology, with cardiac strain biomarkers potentially serving as better indicators of the consequences of local tissue exposure.

Our findings reveal a compelling relationship between overexpression of tryptophan hydroxylase in liver-implanted melanoma cells, serotonin metabolism, and selective right heart valve pathology. The B16F0-Tph1 cells produced excess 5-HT that is clearly detectable in the right ventricular blood but efficiently metabolized before reaching systemic circulation. This compartmentalization of 5-HT exposure explains the selective thickening of right heart valves (pulmonary and tricuspid) while largely sparing left heart valves. The direct correlation between urinary log(5-HT/creatinine) levels and valve volume confirms a dose-dependent relationship, with evidence of a threshold effect at approximately log(5-HT/creatinine) values of 6.5–7.0, above which valve volumes increased more dramatically.

Notably, despite the significant elevation of 5-HT in right heart blood, we found only trace elevations of urinary 5-HT near detection threshold levels, with no corresponding increase in urinary 5-HIAA. In clinical practice, diagnosis of carcinoid syndrome relies primarily on 24-h urinary 5-HIAA measurement, as this metabolite reflects the enzymatic breakdown of excess 5-HT by monoamine oxidase (MAO) and provides a stable indicator of overall 5-HT production by neuroendocrine tumors^26^. This apparent discrepancy can be reconciled by existing literature on mouse-specific serotonin metabolism^27^, which demonstrated that when monoamine oxidase (MAO) pathways are saturated, alternative metabolic routes such as serotonin-O-glucuronidation become dominant. Our findings suggest that the high local 5-HT production overwhelmed the normal MAO pathway, leading to efficient clearance via alternative metabolic pathways with only minimal intact 5-HT appearing in urine.

Mice injected with non-modified B16F0 cells exhibited significantly higher mortality compared to those receiving B16F0-Tph1 cells. This differential survival pattern is consistent with serotonin’s documented anti-proliferative effects on melanoma cells through 5-HT₄ receptor activation^28^, contrasting with its growth-promoting effects in other cancer types^29^. The surviving mice bearing non-modified B16F0 tumors exhibited no evident valve thickening compared to sham controls, indicating that valve remodeling is specifically linked to serotonin production rather than non-specific effects of melanoma cells.

Several limitations warrant discussion. A key limitation is that we measured 5-HT in urine, which reflects systemic levels rather than specifically right ventricular exposure. In previous work, we had measured 5-HT in both the right ventricle and in systemic blood (retro-orbital sinus) and found significant elevation only in the right ventricle. Unfortunately, we could not replicate this approach in the present study since we needed to preserve the right heart integrity for precise valve quantification via HREM. Additionally, while our model successfully reproduces selective right heart involvement, it relies on melanoma rather than neuroendocrine cells. Although this approach enables work with immunocompetent mice, the biological behavior of melanoma cells may not fully replicate the chronic, progressive 5-HT production seen in neuroendocrine tumors. Furthermore, the relatively short exposure duration (5 weeks) likely captures early-stage remodeling, requiring longer-term studies to confirm progression patterns. A key limitation of our study is that HREM signal intensity, while providing sensitive detection of structural heterogeneity, does not directly measure tissue density, fibrosis, or extracellular matrix composition. Definitive evidence of fibrotic or calcific changes will require complementary histological and molecular analyses, which we identify as an important direction for future research. Finally, while HREM provides unprecedented morphological detail, it requires tissue fixation and therefore precludes longitudinal studies in individual animals, limiting our ability to track disease progression dynamically.

Despite these limitations, our mouse model elegantly recapitulates key features of carcinoid heart disease. Our model demonstrates selective right heart valve remodeling, characterized by increased valve volume, thickness, and heterogeneity of grayscale signal intensity. While these findings are consistent with early structural changes seen in carcinoid heart disease, definitive evidence for fibrosis or calcification will require further histological and molecular investigation. The data demonstrate that serotonin functions as a potent stimulus for valvular structural changes, with pathology occurring primarily in right-sided valves due to first-pass exposure before metabolism. The valve-specific remodeling observed directly correlates with urinary 5-HT levels, revealing a clear threshold effect that reinforces the concept that cardiac valve changes require exposure above critical serotonin concentrations. This finding aligns with clinical observations in carcinoid patients, where elevated systemic serotonin alone is highly sensitive (100%) but poorly specific (46%) for predicting carcinoid heart disease, with no clear cutoff point in serum serotonin levels that delineates patients with and without valve involvement^23^.

Our model provides a valuable platform for studying carcinoid heart disease pathogenesis and testing therapeutic interventions. The combination of selective right-heart involvement, precise morphological assessment through HREM, and identification of a 5-HT threshold for valve remodeling creates new opportunities for investigating disease mechanisms. These findings may inform the development of targeted treatments for carcinoid heart disease, potentially focusing on interventions that can be initiated based on urinary biomarkers rather than systemic serotonin levels.

## Methods

### Creation of a 5-HT-producing cell line

B16F0 mouse melanoma cells from American Type Culture Collection (ATCC, USA) were genetically modified to produce 5-HT. The B16F0 line was selected for three key characteristics: (1) its syngeneic nature with C57BL/6 mice, enabling studies in immunocompetent animals, (2) its production of melanin pigments, allowing macroscopic tracking of potential tumor dissemination to the lungs (which could indicate extension to left heart valves), and (3) its established use in tumor growth studies^30–32^. The design, synthesis of the transcript sequence, and generation of the knock-in cell lines were performed by Thermo Fisher Scientific (Carlsbad, USA) using a lentiviral polycistronic vector. The resulting genetically modified murine cell line is referred to as B16F0-Tph1.

The genetic construct (Fig. 1a) was engineered for optimal expression and monitoring capabilities. A CMV promoter was chosen to drive strong, constitutive expression in mammalian cells. The construct contained three key elements in a single reading frame: the TPH1 gene for 5-HT synthesis, a GFP reporter gene for real-time expression monitoring, and a blasticidin resistance gene for stable selection. The expression vector was designed with a P2A self-cleaving peptide sequence between GFP and TPH1 to ensure stoichiometric production of independent proteins from a single transcript, while maintaining stable selection through an independently-promoted blasticidin resistance gene^33^.

Validation of successful genetic modification was performed through multiple complementary approaches. GFP expression was quantified by flow cytometry and confirmed through fluorescence microscopy (Fig. 1b). Functional TPH1 expression was verified by measuring 5-HT production in culture supernatants using HPLC. Comparison between B16F0-Tph1 cells and non-transfected controls revealed significant differences in 5-HT production levels. Based on observed decreases in 5-HT production in later passages, we used cells only between passages 1–2 for all experiments.

### Model development and optimization

Two systematic pilot experiments were conducted to optimize the protocol. The first pilot evaluated tumor burden and survival with varying cell numbers (500–1000–2500–10,000 cells, n = 2 per group). Mice receiving ≥ 2500 cells (n = 4) showed extensive peritoneal carcinomatosis and were euthanized at 4 weeks with intraperitoneal injection of Euthasol 140 mg/kg and death was confirmed by cervical dislocation.

The second pilot determined optimal exposure duration using 1000 cells (n = 3 per group). The melanin pigmentation of B16F0 cells enabled clear macroscopic identification of tumor spread. In this experiment, tumor progression was analyzed as a function of postoperative time: mice operated with the same number of cells were sacrificed sequentially weekly at 5, 6, and 7 weeks postoperatively to establish the kinetics of dissemination of B16F0-Tph1 cells in vivo. In the same experiment, 5-HT was assessed in blood taken from the right ventricle or retro-orbitally (systemic blood) in mice injected with B16F0-Tph1 cells (serotonin-producing melanoma) compared to mice injected with non-modified B16F0 cells (standard melanoma) to verify that the right heart is exposed exclusively to elevated 5-HT compared to systemic circulation in the treated group.

For the main experiment, we selected 1000 cells with 5-week exposure as optimal parameters. Three experimental groups were established: mice injected with B16F0-Tph1 cells (serotonin-producing melanoma), mice injected with non-modified B16F0 cells (standard melanoma), and sham-operated control mice. For the main comparative analyses, the study included 7 B16F0-Tph1 mice and 7 sham-operated controls, with mice randomized across housing units to prevent cage-specific effects. Individual identification was maintained through uniquely numbered ear tags.

The male mice, aged 9 weeks, were sourced from JANVIER LABS and were of the same genetic background as the B16F0 melanocytic cell line. They were housed for 1 week in the animal facility of INSERM, LVTS1148, Bichat and had the same body weight at the start of the experiment (21.1 ± 3.07 g in B16F0-Tph1 vs 22.6 ± 3.3 g in sham mice). Following anesthesia (ketamine 100 mg/kg, xylazine 20 mg/kg), a right subcostal incision exposed the liver. Cell suspension (2 µL PBS) was precisely delivered using a Hamilton® fixed-needle syringe (10 µL, 51 mm, 23 s gauge) at 45° angle to 2 mm depth. To prevent immediate tumor dissemination and ensure hemostasis, Surgicel Fibrillar hemostat (3 × 3 mm) was applied at the injection site. Post-operative care included buprenorphine analgesia (0.1 mg/kg, subcutaneous) and daily monitoring for signs of distress. Blinding was used for each step of the experimental process, and all samples were coded prior to analysis so that the treatment group could not be identified before analysis was completed.

### Ethics

This study was carried out in strict accordance with the recommendations in the Guide for the Care and Use of Laboratory Animals of the National Institutes of Health and in accordance with ARRIVE (Animal Research: Reporting of In Vivo Experiments) guidelines. The protocol was approved by the French Ministry of Education and Research (APAFIS no. 35025) and complies with European Union regulations. Sample size was determined by power calculation (power = 0.8, alpha = 0.05) which indicated a minimum requirement of 7 animals per group. Based on a 40% attrition rate observed in pilot studies, we initially included 10 animals per group to ensure adequate statistical power for the final analysis. All surgery was performed by the same operator, under anesthesia, and all efforts were made to minimize suffering. The humane endpoint criteria established for our experiments were as follows: inactivity, social isolation, arched posture, piloerection, aggressiveness, hind-limb paralysis, weight loss > 20%, skin lesions covering more than 10% of the body, or ascites detected on palpation. Animals exhibiting four or more simultaneous humane endpoint criteria, or any humane endpoint criterion for three consecutive days, were euthanized. Animals presenting fewer than four criteria received immediate analgesic treatment with buprenorphine (0.1 mg/kg, subcutaneously). If humane endpoints were reached, euthanasia was performed the same day. At the conclusion of the experiment, mice were euthanized under deep anesthesia using ketamine (150 mg/kg) and xylazine (30 mg/kg) administered intraperitoneally, followed by cervical dislocation to ensure humane endpoints. All procedures were approved by the institutional animal care committee of INSERM, LVTS1148, Bichat. GS has completed regulatory training in animal experimentation as a project designer "*Concepteurs de projets expérimentaux utilisant des animaux vivants"* at the Paris-Sorbonne University.

### 5-HT Measurement

In our murine model, we measured both urinary 5-HT and 5-HIAA to comprehensively characterize serotonin metabolism, as the mouse model may have different metabolic patterns compared to human patients. Twenty-four hours before planned termination, mice were placed in individual metabolic cages for urine collection. Urine samples were immediately stored at − 20 °C. The analysis was performed at the Biochemistry and Molecular Biology Laboratory of Lariboisière Hospital (Paris, France). Prior to analysis, samples were thawed, centrifuged (2000 g, 10 min, 4 °C), and acidified to pH 2–3. 5-HT and its metabolite 5-HIAA were quantified using High-Performance Liquid Chromatography (HPLC) with electrochemical detection. Chromatographic separation was performed on an Ultimate 3000 HPLC system using a reversed-phase C18 column (150 × 4.6 mm, 5 μm particle size) maintained at 30 °C. The mobile phase consisted of phosphate buffer and methanol (85:15 v/v) at a flow rate of 1.0 mL/min. External standards (50–5000 nmol/L) were used for calibration, and urinary results were normalized to creatinine concentration. All measurements were performed in triplicate to ensure analytical reliability.

The same analytical approach was used to measure 5-HT in cell culture supernatants during the in vitro validation phase as well as in the blood samples collected and processed using identical protocols, except for the creatinine normalization step.

### High-resolution episcopic microscopy

Hearts were arrested in diastole by one-minute immersion in saturated potassium chloride solution and fixed in 4% paraformaldehyde for 24 h. Due to heart length exceeding the standard 5 mm depth resin mold, the apex was removed to focus analysis on the mid-ventricle region and the base containing all four valves. Following PBS washing, samples underwent dehydration through graded ethanol series (30%, 50%, 70%, 90%, 100%, 1 h each) and were embedded in JB-4 resin containing eosin fluorescent dye.

HREM imaging was performed in the Small Animal Histology and Morphology Platform (Necker). Sequential sectioning (1.75 µm) and imaging utilized systematic block-face photography under brightfield. The comprehensive image processing workflow began with initial acquisition of brightfield images at 2048 × 2048 pixels averaging 3 captures per slice for approximately 2000 sections per heart. Image processing continued with registration using ImageJ’s StackReg plugin, followed by background subtraction with a 50-pixel rolling ball algorithm, contrast enhancement at 0.3% saturation, and 3D median filtering with a 2 × 2 × 2 kernel. Final deconvolution in Imaris employed an iterative constrained Richardson-Lucy algorithm with 10 iterations, a signal-to-noise ratio of 20, automatic background correction, and a quality threshold of 0.05.

### Morphometric analysis

Standardized valve measurements were performed using 3D Slicer software (version 4.11) on HREM reconstructions. Three-dimensional reconstruction followed a rigorous protocol beginning with data import and pre-processing of HREM image stacks (2000 sections/heart) into 3D Slicer. Semi-automatic segmentation using threshold-based algorithms was performed, with manual refinement at valve boundaries at a resolution of 10.4 µm (X,Y) × 1.75 µm (Z).

Multi-plane visualization in the Three-dimensional reconstruction of each cardiac valve was illustrated in (Supplementary Figures 2–6) enabled measurement of valve volumes as well as thickness measurements across each cusp/leaflet, from base to free border. Cardiac valve volumes were measured using images taken at 10.4 µm intervals in the X–Y plane and 1.75 µm along the Z axis. Using 3D Slicer 5.8.1 software, we defined regions of interest around each valve by examining the entire deconvoluted heart dataset. We ensured comprehensive inclusion of the target valve through visual inspection and manual addition/exclusion of relevant valve structures. Before quantification, we normalized image intensities across all specimens through histogram matching. The volume measurement involved applying optimized color thresholds to the light channel, followed by voxel counting within each valve region slice-by-slice. The Fill Between Slices tool provided interpolation between manually segmented areas, resulting in volumetric data. Data are expressed as mm^3^. An example of the output is shown in (Supplementary Figure 7).

The thickness measurements included 18 measurements (6 equidistant points from free edge to base for each leaflet/cusp in tricuspid and semilunar valves), and 12 measurements (6 equidistant points per leaflet) in the mitral valve. For measurement validation, inter-observer reliability was established between two independent observers with an intraclass correlation coefficient exceeding 0.90, supported by Bland–Altman analysis. Intra-observer reproducibility was confirmed through repeated measurements at a 1-week interval, yielding a coefficient of variation below 5%. All measurements were taken perpendicular to each leaflet’s long axis (Supplementary Figure 7, an example of measurement points on tricuspid valve), with the first point positioned at valve free edge, the last point at leaflet insertion in the valvular annulus (base), intermediate points equally spaced, and all measurements taken at a 90° angle to leaflet surface. Additional qualitative assessment documented characteristic features of carcinoid heart disease.

For grayscale intensity profiles, valve tissue was assessed using Fiji/ImageJ software (NIH, version 2.9.0). Regions of interest (ROIs) were manually drawn around valve structures in HREM images with consistent selection parameters across all specimens. Intensity profiles were generated using the histogram function, with standardized brightness and contrast settings (window level: 127, window width: 255) applied uniformly across all specimens. Comparative histograms were created to directly visualize the distribution of grayscale intensity values between B16F0-Tph1 and sham control valves. We note that these intensity values reflect optical properties of the tissue and are not direct measures of physical density or ECM composition.

Valve leaflet length and Chordae tendineae dimensions (length and thickness) in the tricuspid valve measurements were performed using 3D Slicer software. The complete contour of each valve leaflet was manually traced from base to free edge, following the natural curvature of the structure. Scale calibration was applied based on the HREM system setting. Total excursion length was measured along the midline of each leaflet/cusp from the annular attachment point to the distal free edge, using consistent anatomical landmarks (ventricular insertion point to distal coaptation margin) across all specimens. Each valve was measured three times independently by two investigators blinded to the experimental groups, and the average measurements were used for analysis.

### Statistical analysis

All statistical analyses and data visualizations were performed using GraphPad Prism (version 9.0, GraphPad Software, San Diego, CA) and JMP (version 15.0, SAS Institute, Cary, NC). After confirming non-normal distribution of valve thickness measurements (D'Agostino-Pearson test), we employed non-parametric methods for group comparisons.

Urinary concentrations of 5-HT and 5-HIAA were normalized to creatinine to account for variations in urine concentration, and these ratios were subsequently log-transformed to reduce data variability and approximate normal distribution. This transformation was particularly effective for the 5-HT/creatinine ratio, reducing the coefficient of variation from over 50% in raw values to approximately 10% in log-transformed data.

Between-group comparisons (Sham vs B16F0-Tph1, n = 7 per group) were conducted using the Kolmogorov–Smirnov test due to its sensitivity to both distribution shape and location differences, which was essential for capturing the heterogeneous pattern of valve remodelling in our model. Kruskal–Wallis tests were used to assess potential asymmetry in valve thickness distribution from free edge to base.

Linear regression analysis was performed to assess the relationship between log-transformed urinary 5-HT/creatinine ratios and right heart valve thickness. The strength of association was determined by the coefficient of determination (R^2^), and significance was assessed at *p* < 0.05. Threshold values for both log(5-HT/creatinine) and valve thickness were calculated using stepwise regression and profiler analysis to identify optimal cutpoints that discriminated between normal and pathological valve states. Diagnostic performance of these thresholds was evaluated by calculating sensitivity and specificity for detecting pathological valve thickening in both pulmonary and tricuspid valves.

For broader correlation analyses, Spearman’s rank correlation was employed to accommodate non-linear associations and potential outliers in the biomarker data. For the threshold effect analysis in the discussion, we employed segmental regression with a data-driven cutpoint at a log(5-HT/creatinine) value of approximately 6.3, based on scatterplot visualization revealing distinct relationship patterns above and below this value.

Data are presented as individual data points with median lines ± IQR to indicate central tendency. Statistical significance was set at *p* < 0.05, with p-values adjusted for multiple comparisons using the false discovery rate method to balance Type I and Type II error risks in this exploratory model characterization.

## Supplementary Information

Below is the link to the electronic supplementary material.

Supplementary Material 1

## Data Availability

The raw data supporting the findings of this study are available from the corresponding author upon reasonable request from qualified researchers.
