# Supplementary material for: Selective right heart valve remodelling in a mouse model of carcinoid disease revealed by high-resolution episcopic microscopy
Source: Sci Rep. 2025 Aug 30;15:31955. doi: 10.1038/s41598-025-16064-8 (PMC12398488; doi:10.1038/s41598-025-16064-8)
Supplement: Supplementary file 1 — Supplementary Material 1 [file 41598_2025_16064_MOESM1_ESM.docx]

**Selective Right Heart Valve Remodelling in a Mouse Model of Carcinoid Disease Revealed by High-Resolution Episcopic Microscopy**

Gaspard Suc*^b^, Mustafa Habib*^b^, Sofiane Mohamed*^a^, Gregory Franck^a^, Samuel Sitbon^b^, Jacques Callebert^c^, Marine Jullien^b^, Baptiste Bazire^b^, Philippe Ruszniewski^d^, Louis de Mestier^d^, Audrey Cailliau^b^, Lydia Deschamps^e^, Dimitri Arangalage^b^, Antonino Nicoletti**^a^, Giuseppina Caligiuri**^b^, Jamila Laschet**^a^

*co-first

** co-last

From:

**^a^ Paris Cité University and Sorbonne Paris Nord University, INSERM, LVTS, F-75018 Paris,** France,

^b^ **Paris Cité University and Sorbonne Paris Nord University, INSERM, LVTS, Cardiology department, AP-HP, Bichat Hospital, F-75018 Paris, France,**

^c^ **Paris Cité University, INSERM U942, Biochemistry and Molecular Biology Department, AP-HP, Lariboisière Hospital, F75010 Paris, France**,

^d^ **Paris Cité University, Pancreatology and Digestive Oncology Departement, AP-HP, Beaujon Hospital, F-92110 Clichy,** France,

^e^ **Pathological anatomy and cytology department, AP-HP, Bichat Hospital, F-75018 Paris,** France

**Word count:** <4500 words (not including Abstract, Methods, References and figure legends)

**Corresponding author:**

Jamila Laschet, PhD,

Inserm LVTS 1148, Bichat Hospital, Paris
Jamila.laschet@inserm.fr

**SUPPLEMENTARY FIGURES 1-9**

| **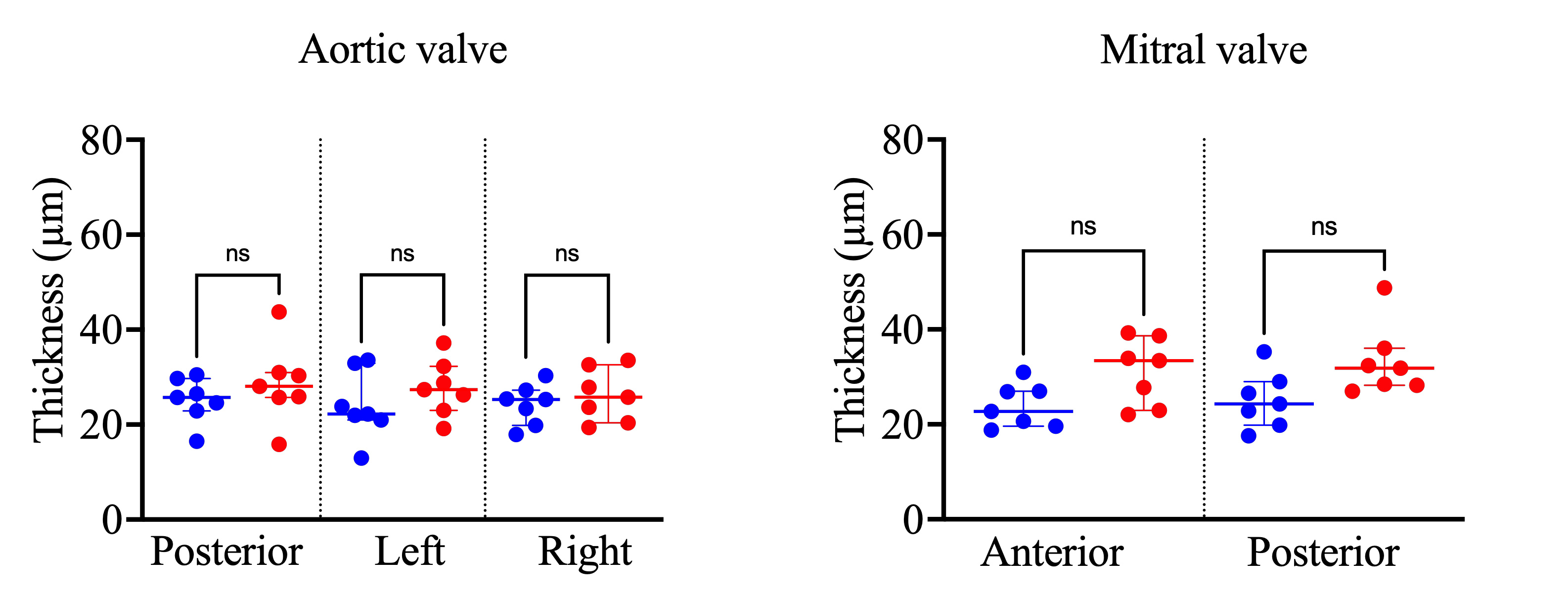** |
| --- |
| **Supplementary Figure 1. Left heart valve thickness remains unchanged between experimental groups.** Aortic valve cusp thickness (left) and mitral valve leaflet thickness (right) showing no significant differences between B16F0-Tph1 mice (red dots) and sham controls (blue dots). **Aortic valve:** Posterior sham: 22.26 ± 11.96 μm, Left (anterior) sham: 25.75 ± 6.81 μm, Right (septal) sham: 25.27 ± 7.42 μm; Posterior B16F0-Tph1: 27.38 ± 9.30 μm, Left B16F0-Tph1: 28.10 ± 5.20 μm, Right B16F0-Tph1: 25.77 ± 12.24 μm (all p > 0.9999). **Mitral valve:** Anterior sham: 22.72 ± 7.39 μm, Posterior sham: 24.33 ± 9.17 μm; Anterior B16F0-Tph1: 33.44 ± 15.68 μm, Posterior B16F0-Tph1: 31.86 ± 7.77 μm (p = 0.1920 and p = 0.2255, respectively). Data are presented as individual data points with median ± IQR; n = 7 per group. Statistical significance was determined using Kolmogorov-Smirnov test (ns: not significant) |

| 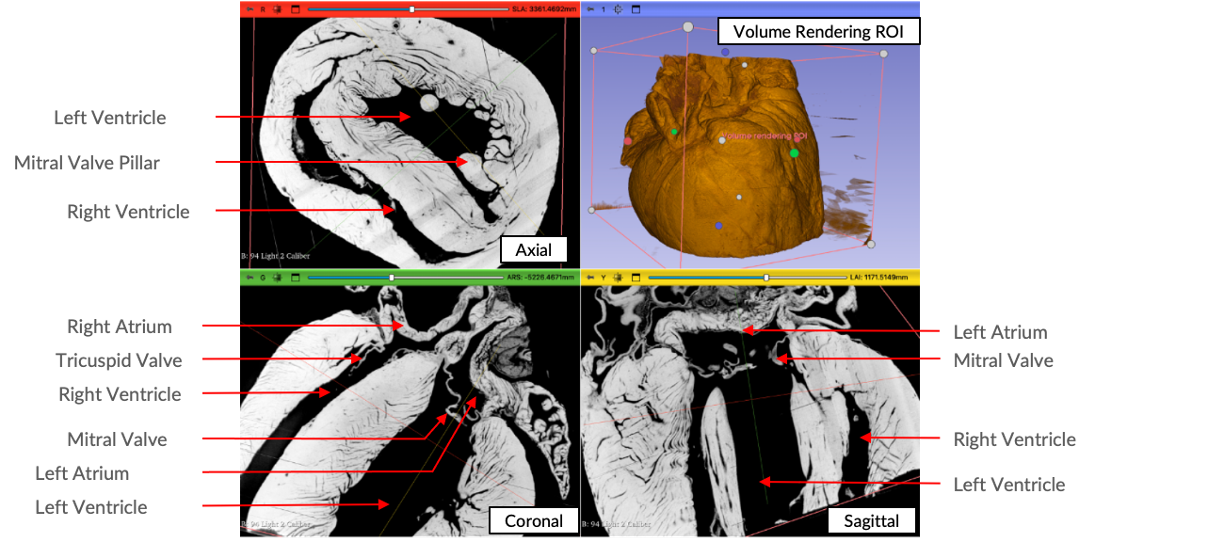 |
| --- |
| **Supplementary Figure 2: Multi-planar 3D reconstruction of cardiac structures from HREM images using 3D Slicer software.** The upper left panel shows an axial view (cross-sectional plane) clearly identifying the left ventricle, mitral valve pillar, and right ventricle, with distinct chamber separation and papillary muscle visualization. The upper right panel displays a volume rendering with region of interest (ROI) highlighting the three-dimensional cardiac architecture. The lower panels demonstrate coronal (left) and sagittal (right) views of the same heart, revealing detailed anatomy of all four cardiac chambers and both atrioventricular valves regardless of their orientation in the original tissue block. This multi-perspective visualization enables standardized assessment of complex three-dimensional structures like cardiac valves that would be challenging to evaluate with conventional histological techniques. |

| 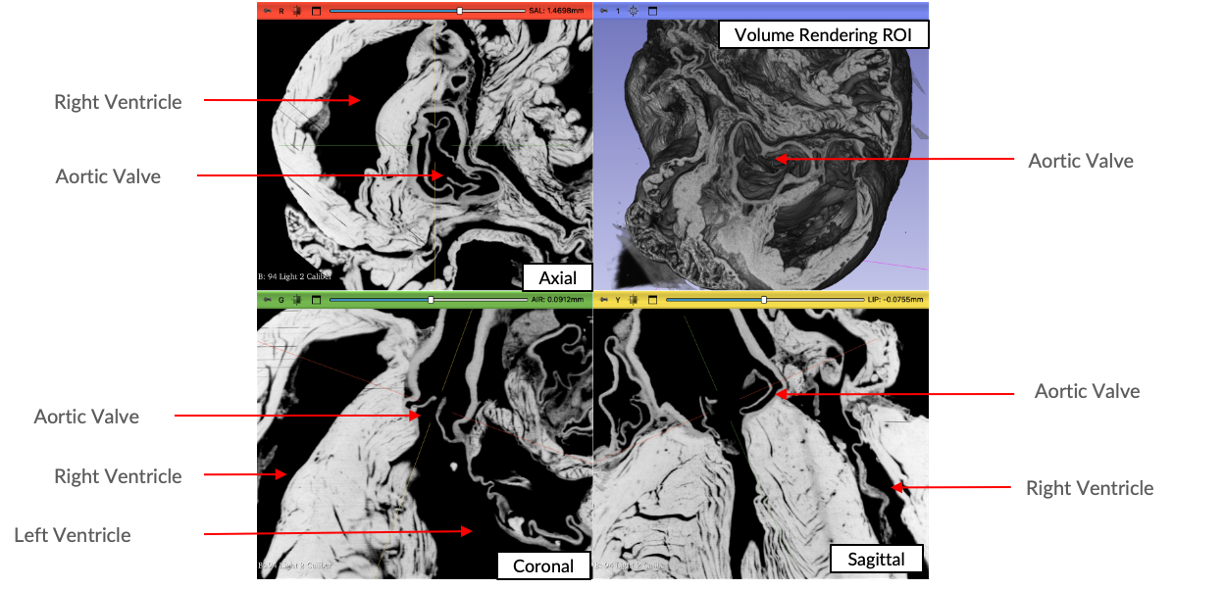 |
| --- |
| **Supplementary Figure 3: Multi-plane visualization of the aortic valve using 3D reconstruction from HREM images.** The upper left panel shows an axial (cross-sectional) view through the aortic valve and right ventricle. The upper right panel presents a tilted cut plane optimized to visualize the aortic valve cusps in their entirety. The lower panels display coronal (left) and sagittal (right) views of the same heart, providing complementary perspectives of the aortic valve in relation to both ventricles. This comprehensive imaging approach enables precise measurement of valve structures regardless of their complex three-dimensional geometry and original orientation within the heart, facilitating standardized quantitative assessment of valve thickness and morphology. |

| **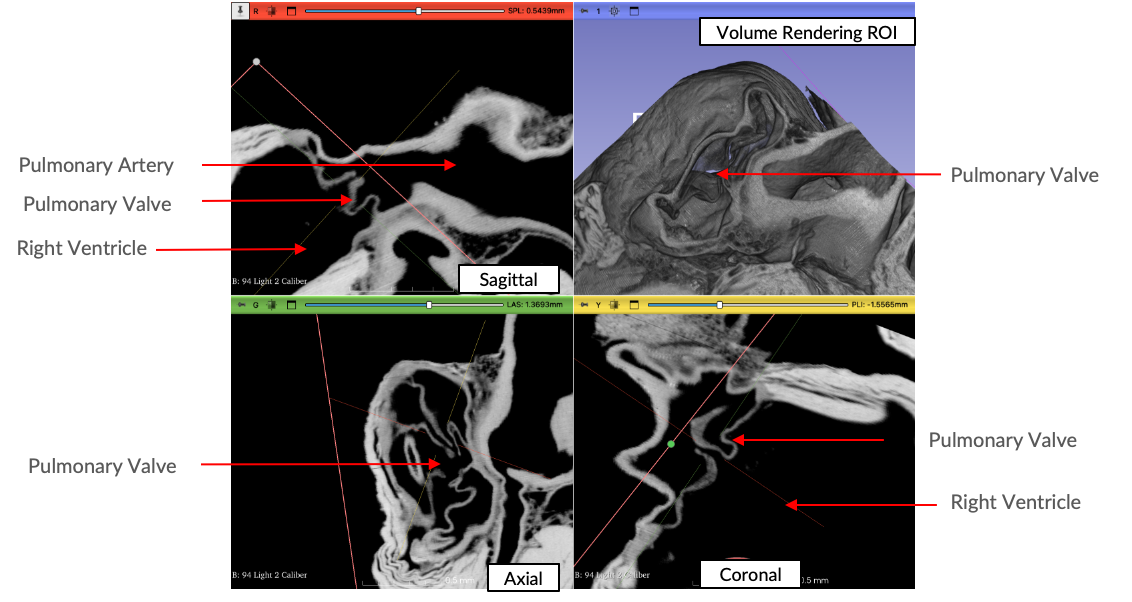** |
| --- |
| **Supplementary Figure 4: Multi-plane visualization of the pulmonary valve and outflow tract using 3D cardiac imaging.** The upper left panel presents a sagittal view depicting the anatomical relationship between the pulmonary artery, pulmonary valve, and right ventricle. The upper right panel shows a volume rendering ROI optimized to visualize the pulmonary valve morphology. The lower panels display axial (left) and coronal (right) views of the same cardiac structures, providing complementary perspectives of the pulmonary valve and its connection to the right ventricle. This comprehensive imaging approach facilitates detailed examination of the pulmonary valve from multiple orientations, enabling precise assessment of valve anatomy and its spatial relationship to surrounding cardiac structures. |

| **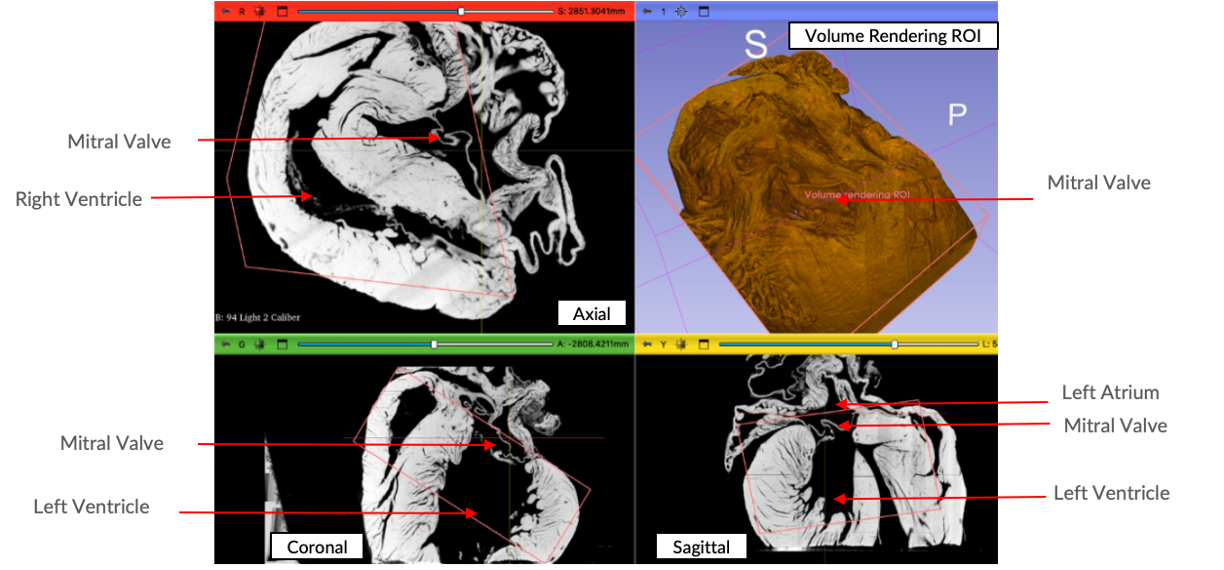** |
| --- |
| **Supplementary Figure 5: Multi-plane visualization of the mitral valve using 3D cardiac imaging.** The upper left panel shows an axial view highlighting the mitral valve and right ventricle. The upper right panel presents a volume rendering ROI with anterior-posterior (P) and superior-inferior (S) orientations, providing a 3D visualization of the cardiac structures with emphasis on the mitral valve. The lower panels display coronal (left) and sagittal (right) views, showing the mitral valve's relationship to the left ventricle and left atrium from complementary perspectives. This comprehensive multi-planar approach enables detailed examination of the mitral valve anatomy and its spatial relationships with surrounding cardiac chambers, facilitating accurate assessment of valve morphology and function. |

| **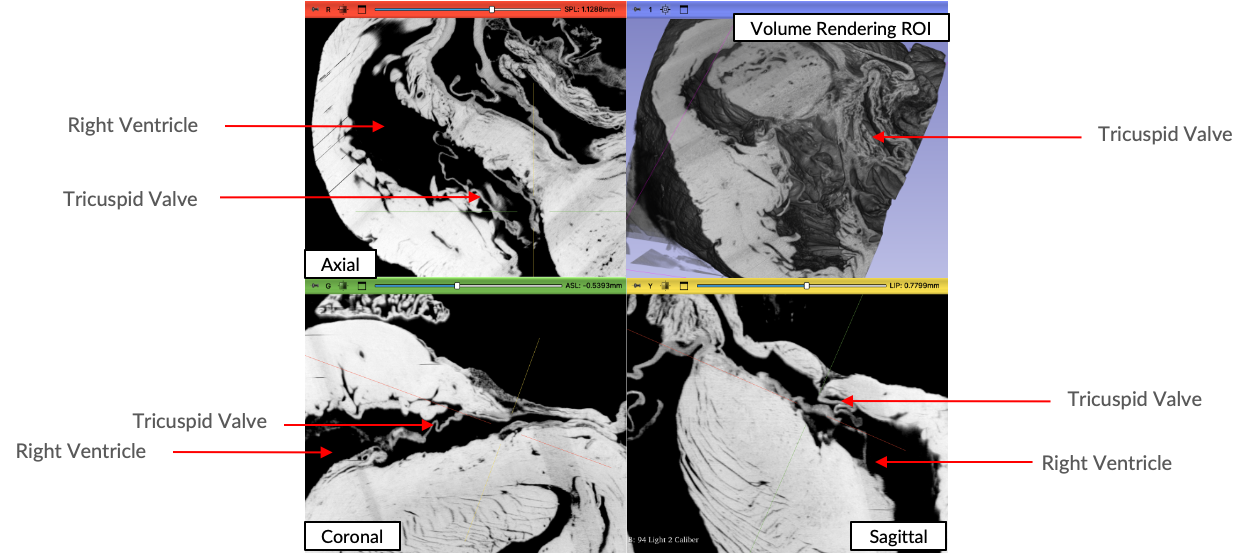** |
| --- |
| **Supplementary Figure 6: Multi-plane visualization of the tricuspid valve using 3D reconstruction from cardiac imaging.** The upper left panel shows an axial (cross-sectional) view of the tricuspid valve and right ventricle. The upper right panel presents a volume rendering ROI optimized to visualize the tricuspid valve structure. The lower panels display coronal (left) and sagittal (right) views of the same heart, providing complementary perspectives of the tricuspid valve in relation to the right ventricle. This comprehensive imaging approach enables precise visualization of the tricuspid valve from multiple angles, facilitating standardized assessment of valve morphology and its anatomical relationship to the right ventricle. |

| A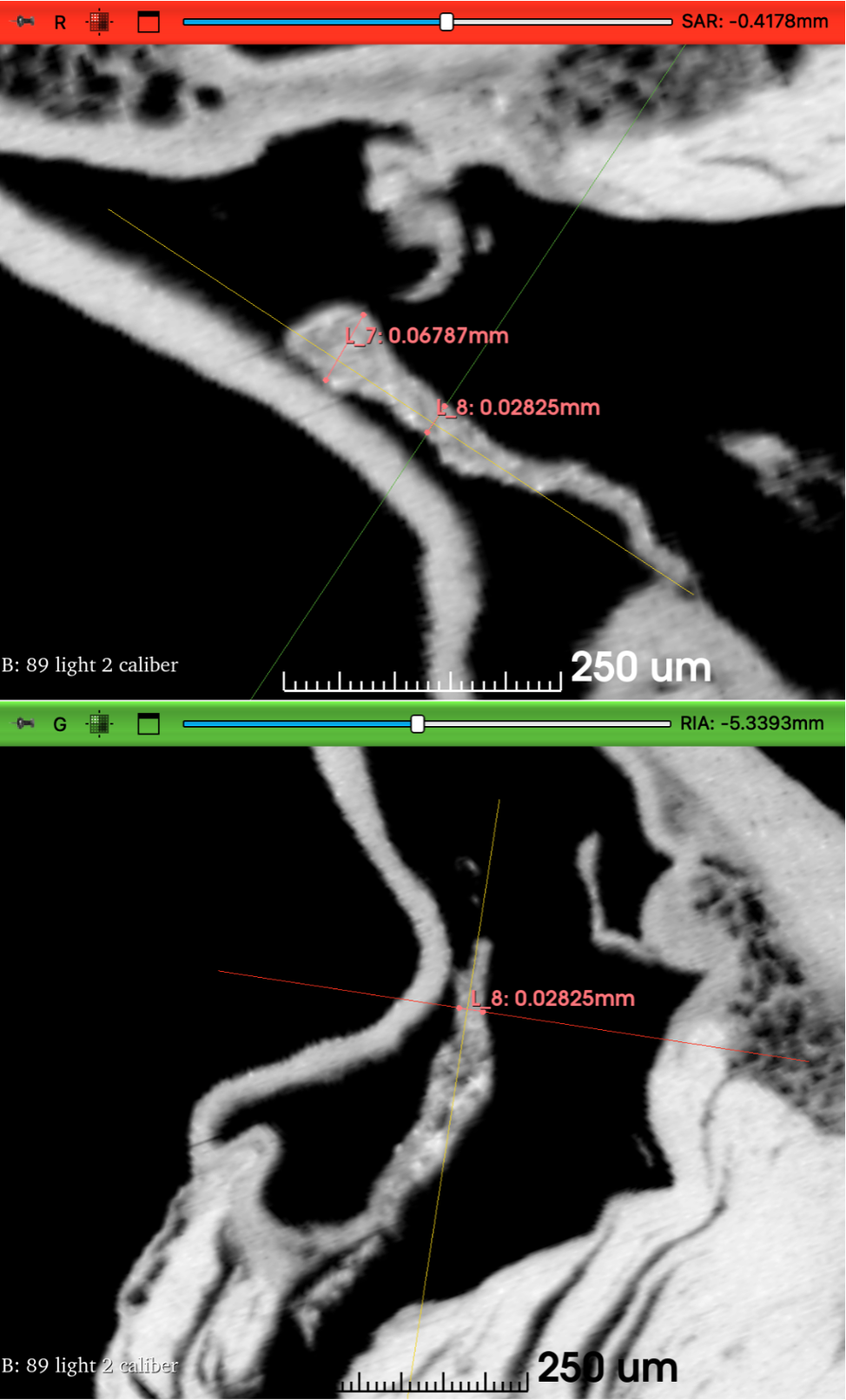 | B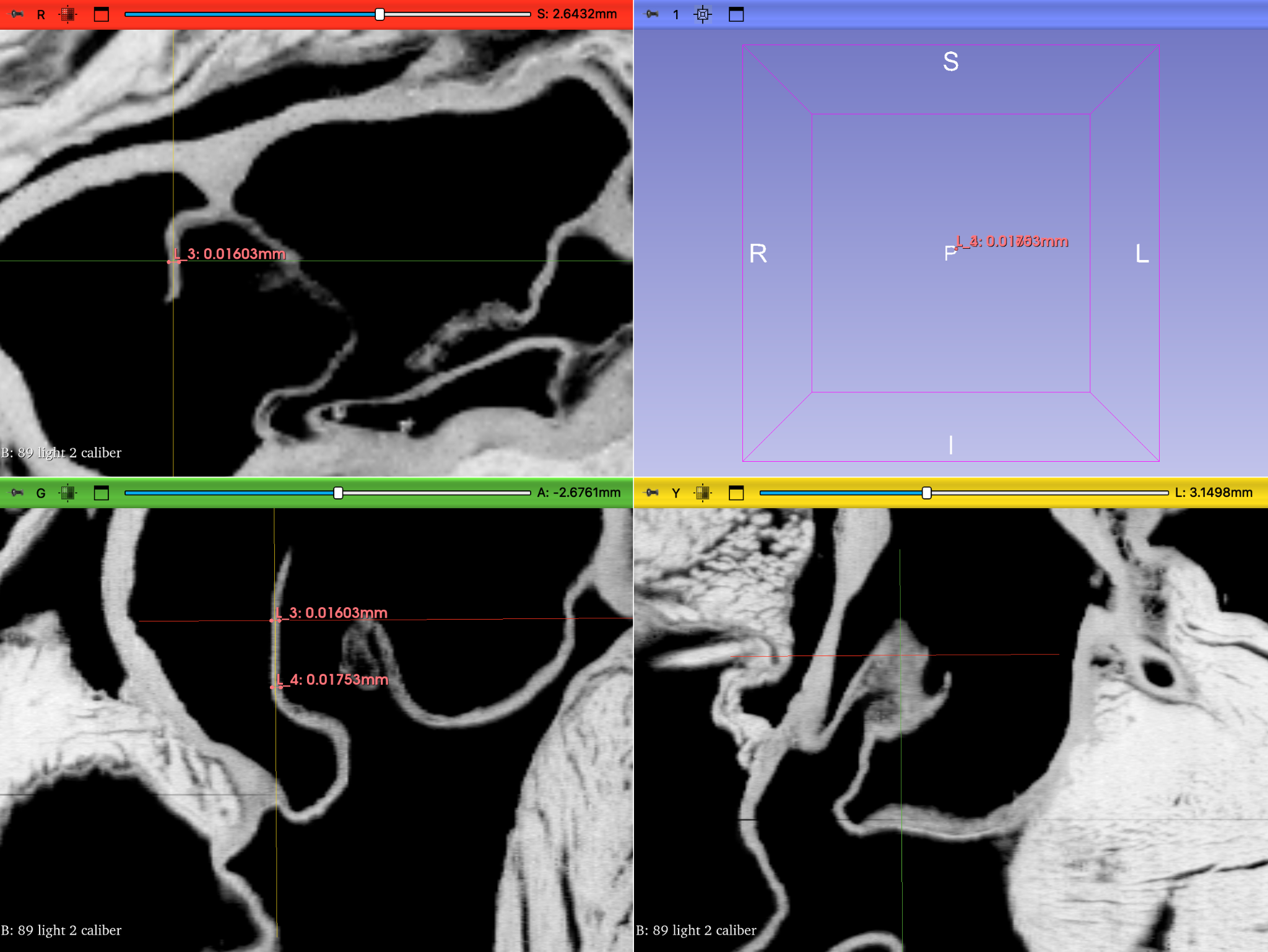 |
| --- | --- |
| **Supplementary Figure 7: Valve thickness measurement methodology.** Standardized thickness measurements were performed perpendicular to the main axis of each valve leaflet. (A) Pulmonary valve and (B) aortic valve from a B16F0-Tph1 mouse demonstrating the measurement approach. Measurements were systematically taken at six equidistant points along each leaflet/cusp, beginning at the free edge and proceeding toward the valve insertion base. The numbering system followed a consistent pattern across all valves: L1-L6 for anterior cusp, L7-L12 for posterior cusp, and L13-L18 for septal cusp. The 3D reconstruction capabilities of HREM allowed dynamic adjustment of the viewing plane, ensuring each measurement maintained perpendicular orientation to the valve's longitudinal axis while preserving consistent spacing between measurement points. Scale bars: 250 μm. | |

| 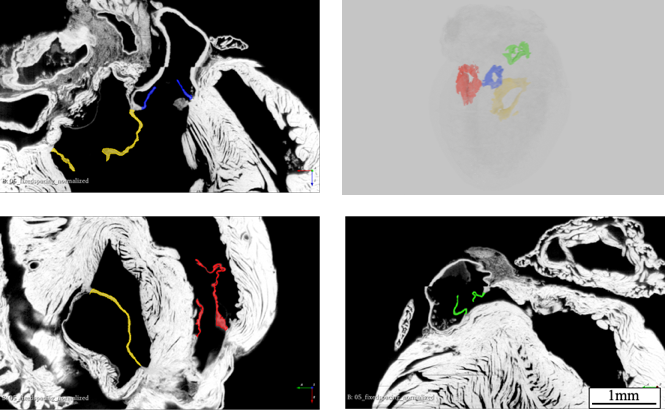 |
| --- |
| **Supplementary Figure 8: Three-dimensional visualization of cardiac valve segmentation.** Multi-perspective HREM imaging of mouse heart with computer-assisted segmentation (3D Slicer software). The four valves are color-coded: aortic valve (blue), mitral valve (yellow), pulmonary valve (green), and tricuspid valve (red). The top right panel shows the 3D volumetric view, while other panels display different sectional orientations. Scale bar: 1 mm. This approach enables precise quantification of valve structures regardless of heart orientation. |

| 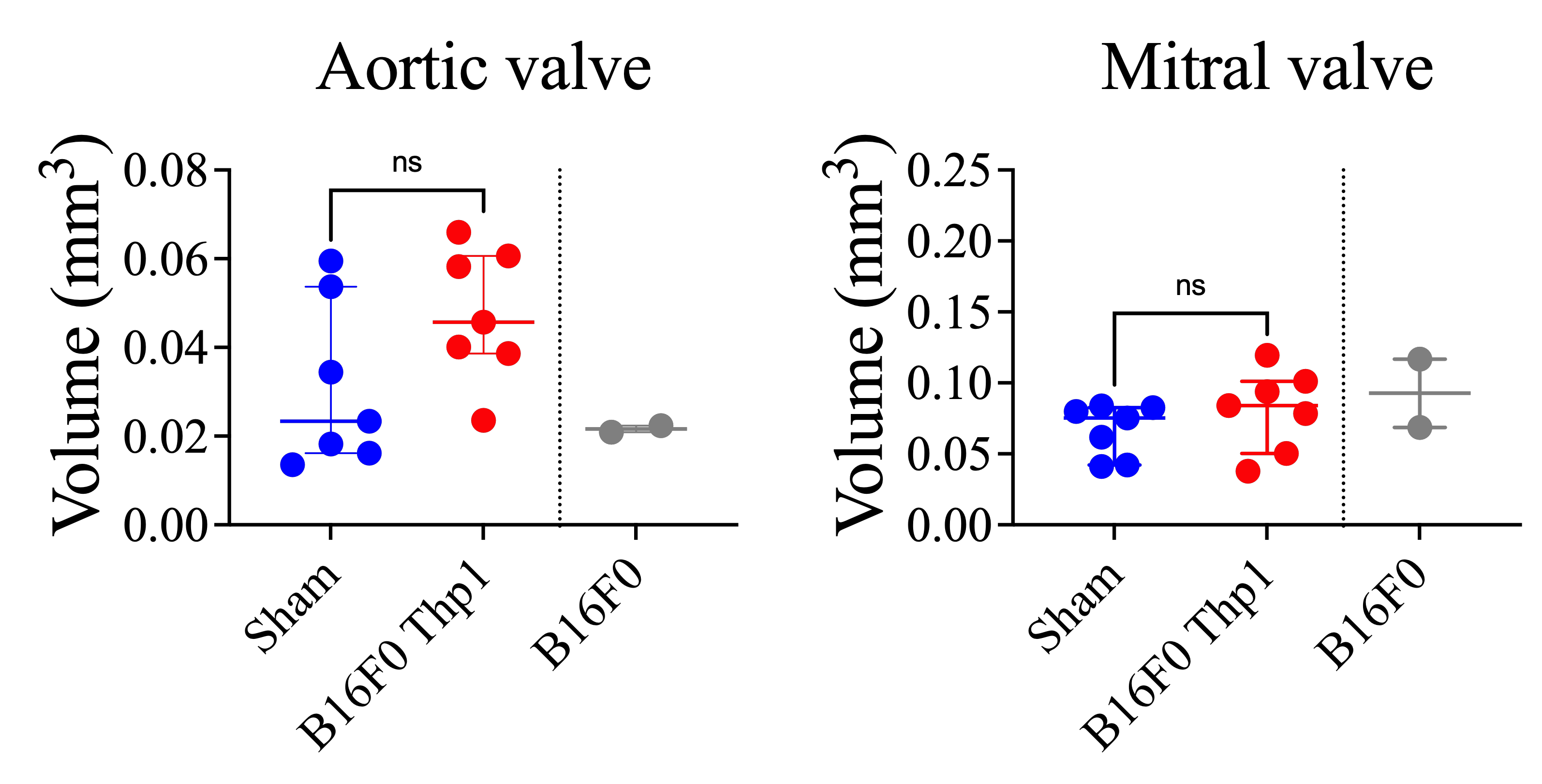 |
| --- |
| **Supplementary Figure 9. Left heart valve volumes remain unchanged between experimental groups.** Volumetric analysis of aortic and mitral valves showing no significant differences between B16F0-Tph1 mice (red dots), sham controls (blue dots), and B16F0 controls (gray dots). **Aortic valve volumes:** Sham: 0.0234 ± 0.0375 mm³, B16F0-Tph1: 0.0457 ± 0.0220 mm³, B16F0: 0.0216 ± 0.0015 mm³ (Sham vs B16F0-Tph1, p = 0.0728). **Mitral valve volumes:** Sham: 0.0754 ± 0.0405 mm³, B16F0-Tph1: 0.0840 ± 0.0508 mm³, B16F0: 0.0927 ± 0.0481 mm³ (Sham vs B16F0-Tph1, p = 0.2593). Data are presented as individual data points with median ± IQR (n = 7 Sham, n = 7 B16F0-Tph1, n = 2 B16F0). Statistical significance was determined using Kolmogorov-Smirnov test (ns: not significant) |
